# Social Stigma in Children with Long COVID

**DOI:** 10.3390/children10091518

**Published:** 2023-09-07

**Authors:** Danilo Buonsenso, Anna Camporesi, Rosa Morello, Cristina De Rose, Matteo Fracasso, Daniela Pia Rosaria Chieffo, Piero Valentini

**Affiliations:** 1 Department of Woman and Child Health, Fondazione Policlinico Universitario A. Gemelli IRCCS, 00168 Rome, Italy; rosa.morello1@guest.policlinicogemelli.it (R.M.); cristina.derose@guest.policlinicogemelli.it (C.D.R.); piero.valentini@unicatt.it (P.V.); 2 Center for Global Health Research Studies, Università Cattolica del Sacro Cuore, 00168 Roma, Italy; 3 Pediatric Anesthesia and Intensive Care Unit, “Vittore Buzzi” Children’s Hospital, 20154 Milan, Italy; anna.camporesi@asst-fbf-sacco.it; 4 Medicine and Surgery, Universityà Cattolica del Sacro Cuore, 00168 Roma, Italy; matteo.fracasso01@icatt.it; 5 Clinical Psychology Unit, Fondazione Policlinico Universitario A Gemelli IRCCS, 00168 Rome, Italy; danielapiarosaria.chieffo@policlinicogemelli.it; 6 Department of Woman, Children and Public Health, Catholic University of Sacred Heart, 00168 Rome, Italy

**Keywords:** long COVID, COVID-19, children, social stigma

## Abstract

There is growing evidence that adults with Long COVID suffer from different sets of stigmata related to their condition. In children with Long COVID, this aspect has never been investigated. This study aims to investigate if children with Long COVID also experience stigma. Methods: Children with a previous SARS-CoV-2 infection evaluated at 3 month follow-ups in a pediatric post COVID unit were asked to fill in an online Long COVID Stigma Scale survey before they were assessed by a pediatrician. Doctors were unaware of children’s responses when they performed a diagnosis of Long COVID or full recovery from previous infection, according to the World Health Organization definition of pediatric Long COVID. Responses to the Stigma scale were then compared in the two cohorts of children. Results: 224 patients responded to the questionnaire; 40 patients were diagnosed with Long COVID. Children with Long COVID significantly more frequently felt embarrassed about having Long COVID (*p* 0.035), felt embarrassed about having physical limitations (*p* < 0.001), felt they were valued less due to Long COVID (*p* 0.003), felt they were different from other peers due to Long COVID (*p* 0.033), felt significantly more frequently that people behaved differently towards them because they might be lying since the diagnosis of Long COVID (*p* 0.006), that they were less respected by others due to Long COVID (*p* 0.017), that other people thought that Long COVID is not a real disease (*p* 0.007), that other people thought that developing Long COVID is a sign of weakness (*p* 0.008), and that other people might judge them negatively due to their diagnosis of Long COVID (*p* < 0.001). Conclusions: Children with Long COVID, similar to adults, are suffering from stigmata due to their condition,. These data may have implication and should be used by the public, policy makers, and healthcare professionals regarding pediatric Long COVID.

## 1. Introduction

Long COVID, or Post COVID Condition, is a subtle multisystemic condition characterized by several long-lasting signs and symptoms that begin with SARS-CoV-2 infection, and have a negative impact on daily life [1]. This condition has been widely described in adults, with evidence of several abnormal events associated with this condition, including chronic vasculitis, dysautonomia, autoimmunity, chronic organ damage, brain hypometabolism, cardiac and thromboembolic events, and others [2,3,4]. Immune biosignatures have also been described [5], as well as post-exertional malaise documented on single or double cardiopulmonary exercise testing [6]. Many of those characteristics are similar to findings from decades of research in patients with myalgic encephalomyelitis/chronic fatigue syndrome (ME/CFS), a post-viral chronic illness that shares many similarities with Long COVID [4].

An important characteristic of ME/CFS is that, historically, despite being described decades ago, this condition is still frequently neglected by doctors globally, and patients suffer continuously from that neglect [7], limiting their possibility to access care and research. Similarly, patients with Long COVID have been reporting that they are also being neglected by the medical community and the public, with little to no access to healthcare systems in several countries. In adults, a pilot study in the United Kingdom found that many patients with Long COVID are experiencing some form of stigma, with 95.4% experiencing at least one type at least ‘sometimes’, and 75.9% experiencing it ‘often’ [8].

In the pediatric population (those younger than 18 years of age), Long COVID has also been described globally [9]. Specifically, the World Health Organization has issued a specific definition of Long COVID for children and adolescents: “Post COVID-19 condition in children and adolescents occurs in individuals with a history of confirmed or probable SARS-CoV-2 infection, when experiencing symptoms lasting at least 2 months which initially occurred within 3 months of acute COVID-19”. Current evidence suggests that the symptoms more frequently reported in children and adolescents with post-COVID-19 condition compared with controls are fatigue, altered smell/anosmia and anxiety. Other symptoms have also been reported [8,9,10]. Symptoms generally have an impact on everyday functioning such as changes in eating habits, physical activity, behaviour, academic performance, social functioning (interactions with friends, peers, family) and achievement of developmental milestones. Symptoms may have a new onset following initial recovery from an acute COVID-19 episode or persist from the initial illness. “They may also fluctuate or relapse over time. Workup may reveal additional diagnoses, but this does not exclude the diagnosis of post COVID-19 condition.” [10,11]. In children, Long COVID seems to have a similar cluster of symptoms and some objective findings have also been documented, including abnormal lung perfusion and ventilation, brain hypometabolism, dysautonomia, and orthostatic intolerance [9]. Nevertheless, members of several family associations related to Long COVID have reported similar episodes of neglect suffered from their children with Long COVID. However, this has not been formally assessed so far.

For those reasons, we performed this study aiming to quantify the burden of stigma experienced in an Italian community-based sample of children and adolescents with lived experience of Long COVID.

## 2. Materials and Methods

### 2.1. Study Design and Operational Definition of Long COVID

This study is a sub-analysis of a prospective cohort of children with microbiologically-confirmed SARS-CoV-2 infection evaluated in person at a referral pediatric post-COVID clinic in Rome, Italy [9]. As previously described, in this outpatient clinic we have followed up children that fully recovered from acute SARS-CoV-2 infection, as well as children with persisting symptoms that have been diagnosed if fulfilled the WHO criteria for Long COVID [10,11]. Long COVID was defined as the persistence of symptoms for at least three months after initial infection, which had a negative impact on daily life, and other possible diagnoses excluded according to the pediatric definitions provided by Stephenson et al. [10] and the WHO [11]. All patients with possible Long COVID underwent tests to exclude the following conditions: anemia, hematologic conditions, coeliac disease, blood glucose, liver and renal function, thyroid problems, autoimmune disease, and other infections, including intestinal parasites. To avoid data contamination, patients identified with alternative diagnoses (e.g., type 1 diabetes, celiac disease) were not classified as Long COVID and excluded from the analysis.

In this paper, we focus on reporting the stigma-related results. Children evaluated in person in our outpatient clinic from 1 January 2023 to 30 April 2023, were asked to fill in an online survey along with their parents (or alone if older than 12 years of age) before being assessed by a pediatrician at the Long COVID clinic. Therefore, the questions were answered with patients blinded to their final diagnosis (recovered from SARS-CoV-2 or having Long COVID). In addition, the evaluating physicians were unaware of the answers to the survey.

### 2.2. Study Tools

To evaluate Long COVID stigma, we adapted a LCSS published and validated in an adult cohort to our pediatric patients [8], which was itself designed following the Health Stigma and Discrimination Framework [12]. The questionnaire was adapted after an internal discussion between our pediatricians and psychologists experienced in the management of children with Long COVID. The questions of this questionnaire were based on existing scales related to other stigmatized chronic conditions, such as Myalgic Encephalomyelitis/Chronic fatigue syndrome (ME/CFS) and HIV [13,14,15,16], and on emerging evidence on stigma suffered by patients with Long COVID [17].

For each family arriving in our outpatient service, before performing the clinical visit, we asked the child and the parents to fill in the questionnaire. The child and the parents were instructed by a doctor on how to complete the questionnaire. For children under the age of 12, the child was invited to reply to the questions by himself/herself, but in the presence of the parents (to provide support or explanation), or directly by the parents in children younger than 5 years of age. For those aged 12 years or older, we invited the child to fill in the question alone, not in presence of parents. For each question, parents and children could reply choosing on a 5-point Likert scale: never, rarely, sometimes, often, and always, coded 1 to 5.

### 2.3. Ethics Committee Approval

The study was conducted in accordance with the Declaration of Helsinki, and approved by the Institutional Review Board (Ethics Committee) of Fondazione Policlinico Universitario A. Gemelli IRCCS (Ethics approval ID4518, Prot0040139/21). Informed consent was obtained from both caregivers of all subjects involved in the study. Our Institution policies include specific informed consent forms adapted for children aged 6 to 12 or 12 and older.

### 2.4. Statistical Analyses

Categorical variables were described as frequencies and percentages, and continuous variables expressed as mean (±standard deviation) or (median; interquartile ranges) as appropriate. The association between answers to the questionnaires and receiving a diagnosis of Long COVID were tested with Pearson’s chi square test and Wilcoxon Rank Sum test. Statistical significance was defined as *p* value ˂ 0.05. Data were analyzed with Stata BE v18.0 (Statacorp LLC, College Station, TX, USA).

## 3. Results

In total, 224 patients (or their caregivers) completed the questionnaire. Mean age was 83.26 (±50) months, median 84 (48–120) months. Patients were categorized as having or not having Long COVID according to clinical evaluation: 40 had Long COVID-age - mean age 119(±41) months, median 114 (90–156) months. Sex was equally distributed and not associated with a diagnosis of Long COVID (*p* 0.11), while children with Long COVID were significantly older than those without (*p* < 0.001).

Table 1 provides details about responses about the entire study population, and divided according to having or not having received a diagnosis of Long COVID. Children with a clinician-based diagnosis of Long COVID had a statistically significant higher probability of living negative social experiences according to most of the investigated areas. In particular, children with Long COVID significantly more frequently felt embarrassed about having Long COVID (*p* 0.035), felt embarrassed about having physical limitations (*p* < 0.001), felt they were valued less due to Long COVID (*p* 0.003), and felt they were different from other peers due to Long COVID (*p* 0.033). In addition, children and adolescents with Long COVID felt significantly more frequently that people behaved differently towards them because they might be lying since the diagnosis of Long COVID (*p* 0.006), that they were less respected by others due to Long COVID (*p* 0.017), that other people thought that Long COVID is not a real disease (*p* 0.007), that other people thought that developing Long COVID is a sign of weakness (*p* 0.008), and that other people might judge them negatively due to their diagnosis of Long COVID (*p* < 0.001).

Patients were then divided according having answered by themselves versus having a parent answer for them in Table 2. Patients whose age was above 12 years were able to answer themselves; they comprised 32 patients, and were significantly associated with a diagnosis of Long COVID, as expected by the older mean age of children with Long COVID. As such, even when we analyzed younger children separately to older children who replied alone, we found higher probability of this older group of children of having encountered negative social experiences. This older group of children had an even higher probability of reporting feeling embarrassed due to Long COVID (*p* 0.01) or physical limitations (*p* 0.004).

## 4. Discussion

In this study, we documented, for the first time to our knowledge, that children with Long COVID experience a number of social issues, including a feeling of isolation, or that they feel that people close to them do not think the disease they suffer from—Long COVID—is a real disease. These findings may have considerable consequence on a child’s mental health and development and should be regarded with attention by physicians and, more in general, by all professionals dealing with children and adolescents.

A recent UK study performed in adults using a similar stigma scale had similar findings, suggesting that the majority of people with Long COVID are experiencing some form of stigma, with 95.4% experiencing at least one type at least ‘sometimes’, and 75.9% experiencing it ‘often’ [8], in line with evidence of stigma associated with other similar chronic conditions such as HIV and myalgic encefphalomyelitis/chronic fatigue syndrome (ME/CFS) [18].

So far, the data on Long COVID stigma have only been only reported in adults [8,17,19,20,21]. Adding evidence of similar events occurring in children is essential to raise awareness around the issue and may serve to find solutions that, in turn, can be translated into clinical practice and healthcare policy. Education about Long COVID among healthcare professionals is one necessary step, but may not be sufficient as history shows us with HIV [8]. It is important that the public is aware of those issues, and efforts to reach a wider audience via advocacy communities should be made [22,23]. In some countries, such as the UK, Long COVID family associations have been formed, which are working hard to inform the public, policy makers, and healthcare professionals, both on the ground and on social media, aiming to create informed and non-judgmental environments for affected patients and their families. Although organizations such as LongCOVIDKids UK have also inspired us to conduct pediatric Long COVID research, including this study, many other countries have not been able to form similar associations, disadvantaging children and adolescents suffering from Long COVID in countries without this type of support.

Of note, a recent study, again performed in adults, found that social stigma in patients with Long COVID is associated with more perceived stress, more depressive symptoms, higher anxiety, and lower mental health related quality of life [24]. Unfortunately, it is not known if social stigma can also affect the mental health of children or adolescents since there are no studies investigating this issue; however, it is plausible to hypothesize that similar negative implications are experienced by younger people as by adults. In adults, there is evidence that feeling stigmatized can impair trust towards healthcare professionals—who themselves can be implicated in the stigmatization of the diseases. Studies have found that people with Long COVID who experienced stigma from healthcare professionals reported losing trust in those professions [25]. Another study reported social stigma experiences in the form of not being taken seriously and being diagnosed correctly [17]. Altogether, these data reinforce the importance of the findings from our study, which aimed to improve awareness on this topic in both the public and healthcare professionals.

Perception of social stigma in children is, anyway, not exclusive to Long COVID, but reported also in children with other chronic conditions or disabilities. Konradi found that the illness experience of children with fibrous dysplasia is impacted by stigma and suggests they should be regularly screened for stigma and psychological distress [26]. Adolescents with chronic pain experience pain-related stigma from medical providers, school personnel, family members, and peers, which may have negative social and health implications [27]. A recent survey found that teens in the general population held beliefs about people with epilepsy that reflected attitudes of stigma [28,29]. Forty percent of the adolescents were not sure if people with epilepsy were dangerous or not, and only thirty-one percent reported that they would date someone with epilepsy [28,29]. All together, these data, along with our study, support the integration of clinical psychologists/therapists in regular pediatric care of patients with Long COVID.

Our findings have to be interpreted according to the large burden that Long COVID seems to have on the pediatric population. Li Jiang and colleagues performed a recent systematic review of persistent clinical features after SARS-CoV-2 in the children [30]. They included published articles and preprints from December 2019 to December 2022 investigating the epidemiology and characteristics of persistent clinical features at least 3 months after COVID-19 in children and adolescents (0–19 years old). The systematic review included 27 cohorts and 4 cross-sectional studies, and involved over 15,000 pediatric participants. A total of more than 20 persistent symptoms and clinical features were reported among children and adolescents. In total, 16.2% (95% confidence interval 8.5% to 28.6%) of the pediatric participants experienced 1 or more persistent symptom(s) at least 3 months post COVID-19. Female gender might be associated with developing certain long COVID symptoms [30]. Although the included studies had great heterogeneity due to significant variations in the definition of “Long COVID”, follow up duration, and methods, the authors concluded that persistent clinical features beyond 3 months among children and adolescents with proven COVID-19 are common and the symptom spectrum is wide. These data are in line with other previous reviews [4]. As such, several children (and their families) are at risk of stigma perception that might affect their quality of life and daily routine. Of note, these numbers do not include children that have developed other post-COVID complications, including Multisystem Inflammatory Syndrome in children (MIS-C, also known as PIMS-TS (Paediatric Inflammatory Multisystem Syndrome temporally related with SARS-CoV-2)), neurological complications, or new diagnosis of type 1 diabetes and other autoimmune diseases.

Another interesting finding is that ratings by patients themselves were worse than those of children that shared responses with or without the support of parents. This finding is difficult to explain, as we did not aim to evaluate reasons behind these discrepancies. Among possible explanations, children may be inhibited by the presence of parents when reporting their symptoms, or the presence of parents may somehow affect (both over- or underestimating) the child’s perceptions.

Our study is not without limitations. First, we involved a relatively small number of children and adolescents with Long COVID. Secondly, the questionnaire was taken from the previous literature in adult populations, and it has not been validated for children. Nevertheless, we did not find a validated tool for this purpose to be used for the pediatric population. Third, stigma is a non-pathological construct and measurements do not have standardized diagnostic criteria, particularly in children. Fourth, a standardized, in-person psychosocial evaluation of these children was not performed; therefore, the real impact of stigma on daily life of these children was not determined. Although we have not evaluated the impact of perceived stigma on daily routine, we speculate that a negative impact on daily life is likely, which deserves to be investigated with dedicated studies. Last, we cannot exclude that, while the cannot exclude onsed, as we did not aim to evaluate reasons behind these discripencies. or anyway the presence of parentobserved stigmata could cause psychosocial problems, the psychological problems themselves may affect the responses or the overall experience Long COVID symptoms. Nevertheless, this study is the first attempt to define perceived stigma in a well-characterized cohort of children that is being prospectively followed up using a rigorous definition of Long COVID [4].

## 5. Conclusions

In conclusion, we documented that children with Long COVID, similar to adults, are suffering from stigma due to their condition, including neglect of their disease. These data have important implications and should not only be used to inform future research projects, but also the public, policy makers and healthcare professionals.

## Figures and Tables

**Table 1:** Responses from the study population, divided according to the final diagnosis of Long COVID made by a blinded pediatrician.

Negative	Total	No Long COVID	Long COVID	*p* Value
	N = 224	N = 184	N = 40	
**Male sex**	120 (53.8%)	103 (56.3%)	17 (42.5%)	0.11
**Age (months)**	84.0 (48.0–120.0)	72.0 (36.0–108.0)	114.0 (90.0–156.0)	<0.001
**I felt embarrassed due to Long COVID**				0.035
1 (never)	193 (86.2%)	164 (89.1%)	29 (72.5%)	
2	16 (7.1%)	11 (6.0%)	5 (12.5%)	
3	10 (4.5%)	5 (2.7%)	5 (12.5%)	
4	4 (1.8%)	3 (1.6%)	1 (2.5%)	
5 (always)	1 (0.4%)	1 (0.5%)	0 (0.0%)	
**I felt embarrassed due to my physical limitation**				<0.001
1	194 (86.6%)	169 (91.8%)	25 (62.5%)	
2	18 (8.0%)	11 (6.0%)	7 (17.5%)	
3	8 (3.6%)	2 (1.1%)	6 (15.0%)	
4	3 (1.3%)	2 (1.1%)	1 (2.5%)	
5	1 (0.4%)	0 (0.0%)	1 (2.5%)	
**I felt less valued due to Long COVID**				0.003
1	199 (88.8%)	170 (92.3%)	29 (72.5%)	
2	15 (6.7%)	8 (4.4%)	7 (17.5%)	
3	6 (2.7%)	4 (2.2%)	2 (5.0%)	
4	4 (1.8%)	2 (1.1%)	2 (5.0%)	
**I felt different due to Long COVID**				0.033
1	199 (88.8%)	169 (91.8%)	30 (75.0%)	
2	13 (5.8%)	7 (3.8%)	6 (15.0%)	
3	7 (3.1%)	5 (2.7%)	2 (5.0%)	
4	3 (1.3%)	2 (1.1%)	1 (2.5%)	
5	2 (0.9%)	1 (0.5%)	1 (2.5%)	
**Due to Long COVID, people are uncomfortable with me**				0.15
1	203 (90.6%)	168 (91.3%)	35 (87.5%)	
2	13 (5.8%)	11 (6.0%)	2 (5.0%)	
3	5 (2.2%)	2 (1.1%)	3 (7.5%)	
4	1 (0.4%)	1 (0.5%)	0 (0.0%)	
5	2 (0.9%)	2 (1.1%)	0 (0.0%)	
**Due to Long COVID, people are rude to me**				0.43
1	208 (92.9%)	172 (93.5%)	36 (90.0%)	
2	7 (3.1%)	6 (3.3%)	1 (2.5%)	
3	3 (1.3%)	2 (1.1%)	1 (2.5%)	
4	4 (1.8%)	2 (1.1%)	2 (5.0%)	
5	2 (0.9%)	2 (1.1%)	0 (0.0%)	
**Close people did not want spent time with me anymore when they knew I may have Long COVID**				0.096
1	215 (96.0%)	176 (95.7%)	39 (97.5%)	
2	7 (3.1%)	7 (3.8%)	0 (0.0%)	
3	1 (0.4%)	0 (0.0%)	1 (2.5%)	
5	1 (0.4%)	1 (0.5%)	0 (0.0%)	
**People thought I was lying about my health**				0.006
1	212 (94.6%)	177 (96.2%)	35 (87.5%)	
2	6 (2.7%)	5 (2.7%)	1 (2.5%)	
3	3 (1.3%)	1 (0.5%)	2 (5.0%)	
4	2 (0.9%)	0 (0.0%)	2 (5.0%)	
5	1 (0.4%)	1 (0.5%)	0 (0.0%)	
**People stopped respecting me due to Long COVID**				0.017
1	214 (95.5%)	177 (96.2%)	37 (92.5%)	
2	5 (2.2%)	5 (2.7%)	0 (0.0%)	
3	4 (1.8%)	1 (0.5%)	3 (7.5%)	
5	1 (0.4%)	1 (0.5%)	0 (0.0%)	
**People do not think Long COVID is a real disease**				0.007
1	178 (79.5%)	153 (83.2%)	25 (62.5%)	
2	17 (7.6%)	11 (6.0%)	6 (15.0%)	
3	17 (7.6%)	14 (7.7%)	3 (7.5%)	
4	5 (2.2%)	3 (1.6%)	2 (5.0%)	
5	7 (3.1%)	3 (1.6%)	4 (10.0%)	
**People think having Long COVID is a sign of weakness**				0.008
1	199 (88.8%)	168 (91.3%)	31 (77.5%)	
2	9 (4.0%)	7 (3.8%)	2 (5.0%)	
3	6 (2.7%)	3 (1.6%)	3 (7.5%)	
4	4 (1.8%)	1 (0.5%)	3 (7.5%)	
5	6 (2.7%)	5 (2.7%)	1 (2.5%)	
**I feel I can be judged negatively due to Long COVID**				<0.001
1	204 (91.1%)	174 (94.6%)	30 (75.0%)	
2	8 (3.6%)	6 (3.3%)	2 (5.0%)	
3	8 (3.6%)	2 (1.1%)	6 (15.0%)	
4	1 (0.4%)	0 (0.0%)	1 (2.5%)	
5	3 (1.3%)	2 (1.1%)	1 (2.5%)	

**Table 2:** Comparisons of responses according to patients have self or non-self answers.

	Total	No Self-Answer	Self-Answer	*p* Value
		N = 192	N = 32	
**I felt embarrassed due to Long COVID**				**0.010**
1 (never)	193 (86.2%)	170 (88.5%)	23 (71.9%)	
2	16 (7.1%)	13 (6.8%)	3 (9.4%)	
3	10 (4.5%)	7 (3.6%)	3 (9.4%)	
4	4 (1.8%)	2 (1.0%)	2 (6.2%)	
5 (always)	1 (0.4%)	0 (0.0%)	1 (3.1%)	
**I felt embarrassed due to my physical limitation**				**0.004**
1	194 (86.6%)	171 (89.1%)	23 (71.9%)	
2	18 (8.0%)	14 (7.3%)	4 (12.5%)	
3	8 (3.6%)	6 (3.1%)	2 (6.2%)	
4	3 (1.3%)	1 (0.5%)	2 (6.2%)	
5	1 (0.4%)	0 (0.0%)	1 (3.1%)	
**I felt less valued due to Long COVID**				**0.057**
1	199 (88.8%)	174 (90.6%)	25 (78.1%)	
2	15 (6.7%)	12 (6.2%)	3 (9.4%)	
3	6 (2.7%)	3 (1.6%)	3 (9.4%)	
4	4 (1.8%)	3 (1.6%)	1 (3.1%)	
**I felt different due to Long COVID**				**0.36**
1	199 (88.8%)	173 (90.1%)	26 (81.2%)	
2	13 (5.8%)	11 (5.7%)	2 (6.2%)	
3	7 (3.1%)	5 (2.6%)	2 (6.2%)	
4	3 (1.3%)	2 (1.0%)	1 (3.1%)	
5	2 (0.9%)	1 (0.5%)	1 (3.1%)	
**Due to Long COVID people, are uncomfortable with me**				**0.45**
1	203 (90.6%)	174 (90.6%)	29 (90.6%)	
2	13 (5.8%)	12 (6.2%)	1 (3.1%)	
3	5 (2.2%)	3 (1.6%)	2 (6.2%)	
4	1 (0.4%)	1 (0.5%)	0 (0.0%)	
5	2 (0.9%)	2 (1.0%)	0 (0.0%)	
**Due to Long COVID, people are rude to me**				**0.81**
1	208 (92.9%)	179 (93.2%)	29 (90.6%)	
2	7 (3.1%)	6 (3.1%)	1 (3.1%)	
3	3 (1.3%)	2 (1.0%)	1 (3.1%)	
4	4 (1.8%)	3 (1.6%)	1 (3.1%)	
5	2 (0.9%)	2 (1.0%)	0 (0.0%)	
**Close people did not want spent time with me anymore when they knew I may have Long COVID**				**0.95**
1	215 (96.0%)	184 (95.8%)	31 (96.9%)	
2	7 (3.1%)	6 (3.1%)	1 (3.1%)	
3	1 (0.4%)	1 (0.5%)	0 (0.0%)	
5	1 (0.4%)	1 (0.5%)	0 (0.0%)	
**People thought I was lying about my health**				**0.040**
1	212 (94.6%)	183 (95.3%)	29 (90.6%)	
2	6 (2.7%)	6 (3.1%)	0 (0.0%)	
3	3 (1.3%)	1 (0.5%)	2 (6.2%)	
4	2 (0.9%)	1 (0.5%)	1 (3.1%)	
5	1 (0.4%)	1 (0.5%)	0 (0.0%)	
**People stopped respecting me due to Long COVID**				**0.006**
1	214 (95.5%)	186 (96.9%)	28 (87.5%)	
2	5 (2.2%)	4 (2.1%)	1 (3.1%)	
3	4 (1.8%)	1 (0.5%)	3 (9.4%)	
5	1 (0.4%)	1 (0.5%)	0 (0.0%)	
**People do not think Long COVID is a real disease**				**<0.001**
1	178 (79.5%)	158 (82.3%)	20 (62.5%)	
2	17 (7.6%)	15 (7.8%)	2 (6.2%)	
3	17 (7.6%)	12 (6.2%)	5 (15.6%)	
4	5 (2.2%)	5 (2.6%)	0 (0.0%)	
5	7 (3.1%)	2 (1.0%)	5 (15.6%)	
**People think having Long COVID is a sign of weakness**				**0.002**
1	199 (88.8%)	174 (90.6%)	25 (78.1%)	
2	9 (4.0%)	8 (4.2%)	1 (3.1%)	
3	6 (2.7%)	4 (2.1%)	2 (6.2%)	
4	4 (1.8%)	4 (2.1%)	0 (0.0%)	
5	6 (2.7%)	2 (1.0%)	4 (12.5%)	
**I feel I can be judged negatively due to Long COVID**				**<0.001**
1	204 (91.1%)	180 (93.8%)	24 (75.0%)	
2	8 (3.6%)	5 (2.6%)	3 (9.4%)	
3	8 (3.6%)	6 (3.1%)	2 (6.2%)	
4	1 (0.4%)	1 (0.5%)	0 (0.0%)	
5	3 (1.3%)	0 (0.0%)	3 (9.4%)	

## Data Availability

Data are available upon request to the corresponding author.
